# Posterior tibial nerve stimulation versus desmopressin in treating children with primary mono-symptomatic nocturnal enuresis. A randomized clinical trial

**DOI:** 10.1080/20905998.2024.2373404

**Published:** 2024-07-10

**Authors:** Mohamed Abdelghany, Mahmoud S Amar, Ahmed I Shoukry, Hany Morsi, Hesham I Mohamed

**Affiliations:** Urology Department, Cairo University Hospital, Cairo, Egypt

**Keywords:** Nocturnal enuresis, PTNS, desmopressin

## Abstract

**Objective:**

Assessment of the efficacy of Posterior Tibial Nerve Stimulation (PTNS) versus Desmopressin in treating Primary Mono-symptomatic Nocturnal Enuresis (PMNE).

**Patients and methods:**

This randomized clinical trial was conducted at the Urology department of Abo Elreesh pediatric hospital, Cairo University on 80 children, aged between 5 and 13 years old, diagnosed to have PMNE between June 2020 and November 2020. Children were divided into two equal groups; those who underwent PTNS (as one session per week for 12 weeks) (Group A) and those who received Desmopressin 0.2 mg. single evening dose for 12 weeks (Group B). Both groups were constructed to adhere to behavioral therapy and were statistically evaluated regarding the frequency of nocturnal enuresis (NE) before, after treatment, and after 1 month of follow-up.

**Results:**

Both groups showed statistically significant improvement in the frequency of NE before and after treatment (*p* < 0.001), but there were no statistically significant differences between them (*p* = 0.763). There was a statistically significant relapse of NE frequency after 1 month of follow-up after completion of treatment in both groups (*p* < 0.001), with no statistically significant differences between the two groups (*p* = 0.075).

**Conclusion:**

Posterior tibial nerve stimulation and Desmopressin are viable treatment options for children with primary mono-symptomatic nocturnal enuresis. However, relapse in some responders with time suggests the need for maintenance therapy.

## Introduction

Primary mono-symptomatic nocturnal enuresis (PMNE) is a prevalent problem with a prevalence of 15–20% at the age of five [1]. It is defined as NE, without daytime symptoms of bladder dysfunction, and the child has never been continuously dry for at least 6 months [2]. PMNE is a heterogeneous condition for which different causative factors have been advocated, such as: sleep disturbances, nocturnal polyuria, reduced bladder capacity or urinary bladder dysfunction, and upper airway obstruction [3]. Children with PMNE often have normal nocturnal bladder storage but some of them have nocturnal OAB [3]. Although it is a benign condition, it causes substantial psycho-social distress to both children and their families, especially if not treated [2]. There needs to be a consensus regarding the proper management of children with PMNE. The currently recommended treatment modalities include behavioral therapy, alarm system, medical treatment with desmopressin with or without anticholinergics (In cases resistant to desmopressin alone or if there is a suspicion for night time OAB) [4]. Neither of them is effective in all children and is usually associated with a significant relapse rate [5,6]. The pathogenesis of refractory NE may be attributed to decreased bladder capacity and/or OAB [7,8]. Transcutaneous electrical nerve stimulation, through neuromodulation, has shown promising results on the normalization of incontinence frequency [9–13]. Clinical studies have shown that this method can modulate excitatory and inhibitory components of lower urinary tract function, which was noticed in urodynamic parameters [9–13]. Posterior tibial nerve stimulation was introduced as a neuro-modulation therapy for NE with early promising results, especially in refractory cases [14]. Several systematic reviews and randomized trials have documented the potential benefits of electrical neural stimulation for PMNE. However, the quality of the included studies was low and different types of electrical neuromodulation have been used [15–17]. Some studies showed promising results of PTNS usage in treating PMNE especially in refractory cases [14] while others claimed that there is no anti-enuretic effect of transcutaneous electrical nerve stimulation in children with PMNE when compared to placebo [9].

## Materials and methods

This randomized clinical trial was conducted at the Urology department of Abo Elreesh pediatric hospital, Cairo University on 80 children aged between 5 and 13 years old and diagnosed to have PMNE between June 2020 and November 2020. We chose this age limit as EAU 2024 guidelines, pediatric urology section, strongly recommended not to treat children less than 5 years of age in whom spontaneous cure is likely [18]. The upper limit chosen was 13 years as this is the upper limit of age that our institute (Abo Elreesh pediatric hospital) allows. The Cairo university Research Ethics Committee (REC) reviewed and approved the study protocol. Informed written consent was obtained from the parents of all children in this trial.

Eighty children aged between 5 and 13 years old were presented with more than two episodes of bedwetting per week (not improved with behavioral therapy alone for at least 1 month), who had not been dry for at least months (primary cases only), with otherwise normal physical examination, normal urine analysis, and normal urinary tract ultrasound were included in this trial. While children with diurnal enuresis, secondary NE, Fecal soiling, Urinary tract infection, cardiovascular, renal, or neurological disorders and those on other treatments were excluded from this trial.

All children provided a detailed history (regarding the severity of NE using a nocturnal enuresis diary for the last 2 weeks), family history and underwent complete physical examination. Urine analysis, kidney functions, and urinary ultrasound.

The clinical severity of NE was defined as Infrequent (1–2 wetting episodes per week), Moderately severe (3–5 wetting episodes per week), or Severe (6–7 wetting episodes per week) [19].

Children were randomly divided into two equal groups by the Concealed random allocation method for randomization.

Group A: (40 children): underwent 12 weekly sessions of PTNS using the Urgent® PC Neuromodulation System. After administration of a topical anesthetic agent, A 34-gauge stainless steel needle is inserted percutaneously about three finger breadths cephalad to the medial malleolus and 1 cm from the posterior margin of the tibia at an angle of 60 degrees from the skin surface. The negative electrode was placed on the same leg near the foot’s arch. The needle and the electrode were connected to a low voltage (9 V) stimulator (Urgent® PC Neuromodulation System). The device was turned on, and amplitude was slowly increased until flexion of the big toe or fanning of other toes occurred. Each session continued for 30 min [13]. The duration of treatment in trials which study the effectiveness of PTNS in treatment of PMNE was from 6 to 12 weeks [9,12,14].

Group B (40 children) were given medical treatment (Desmopressin 0.2 mg, oral tablets, single evening dose) for 12 with tapering to half of the daily dose for 2 weeks before discontinuation.

Both groups were constructed to continue adherence to behavioral therapy, including (Fluid restriction at night, complete bladder emptying before sleep, and Awakening 2 h after sleep to void). The two groups were compared regarding the frequency of nocturnal enuresis before, after treatment, and after 1 month of follow-up.

Clinical improvement of NE severity was defined as downstaging of severity while remaining stationary or upstaging was defined as worsening (e.g. child with moderately severe NE) who had either infrequent bed wetting or became completely dry (after treatment) is considered improvement. If this child had severe or moderately severe NE after treatment, it was considered as worsening.

## Statistical analysis

Statistical analysis was performed using SPSS version 21. Qualitative data was presented by number and percentage; age was presented by mean, standard deviation, and range. Statistical tests done for parametric quantitative data were student’s t test, non-parametric data chi-square test, Fischer exact test for independent qualitative data, and Friedman test for multiple related qualitative data. The level of significance was considered if p was equal to or below 0.05.

### Sample size

Using power and sample size calculator for comparing two proportions; with 0.05 alpha error and power of the study 0.80. Calculating minimal sample size needed to study efficacy of posterior tibial nerve stimulation in the treatment of children with primary mono-symptomatic nocturnal enuresis. Based on the literature, the improvement proportion with medical treatment was 76% (Onol et al. [19]) and 78.6% with the posterior tibial nerve stimulation (Raheem et al. [14]), with 0.25 non inferiority or non-superiority margin. The total sample size calculated was 80 children (40 for posterior tibial nerve stimulation and 40 for conventional medical treatment).

**Table 1.** Patient characteristics.

		Group A (n = 40)	Group B (n = 38)	P value
Age mean (y)		8.62 ± 2.4474	9.135 ± 2.536	0.184**
Gender	Males	22(55%)	18(47.4%)	0.201*
	Females	18(45%)	20(52.6%)	
Family history	Positive	3(7.5%)	3 (7.9%)	0.638*
	Negative	37 (92.5%)	35 (92.1%)	
severity of NE before treatment	severe	28(70%)	23(60.5%)	0.77*
	Moderately severe	12(30%)	15(39.5%)	

*chi-square test.

**student t-test.

Also, there was no statistically significant difference between the two groups regarding the severity of NE before treatment (*p* = 0.77).

**Figure 1.**
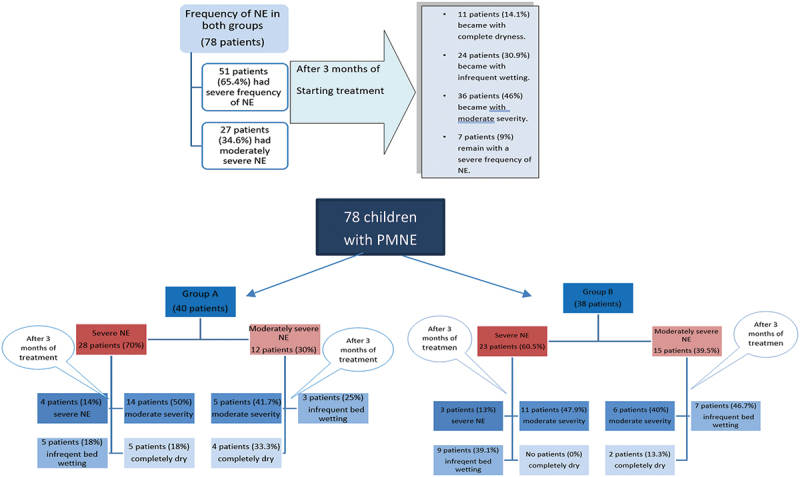
Effect of 3 months of treatment in both groups.

Both groups showed statistically significant improvement in the frequency of NE before and after treatment (*p* < 0.001) (Figure 1).

Both groups; there were no statistically significant differences regarding improvement in the frequency of NE (*p* = 0.763); (Table 2)

**Table 2.** Improvement in frequency of NE after 3 months of treatment.

Groups			Frequency of NE just after starting treatment for 3 months			Total	P value
			Not improved	Improved	Worsened		
Group A (*n*=40)	Frequency of NE before starting treatment	severe	4(14.3%)	24(85.7%)	0(0.0%)	28	0.763*
		moderately severe	5(41.7%)	7(58.3%)	0(0.0%)	12	
	Total		9(22.5%)	31(77.5%)	0(0.0%)	40	
Group B (*n*=38)	Frequency of NE before starting treatment	severe	3(13%)	20(87%)	0(0.0%)	23	
		moderately severe	6(40%)	9(60%)	0(0.0%)	15	
	Total		9(23.7%)	29(76.3%)	0(0.0%)	38	

*chi-square test.

## Results

Eighty children with a mean age of 8.9 ± 2.5 years (aged from 5 to 13 years old) were included in this study. Fifty-one percent of them were males, while the others were females.

Children of the study were divided into two groups, A and B (40 children for each), (within group B, two children discontinued the study because their parents found that there was no improvement). There was no statistically significant difference regarding either age, gender, or family history of NE of children between the two groups (*p* = 0.184, 0.201, and 0.192, respectively). (Table 1)

**Figure 2.**
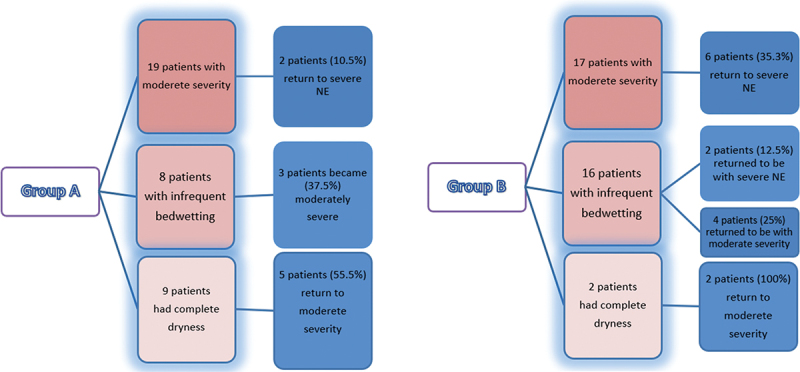
Relapse in both groups.

After 1 month of follow-up after the completion of treatment in both groups, there was a statistically significant relapse of NE frequency in both groups (*p* < 0.001) with no statistically significant difference between the groups (*p* = 0.075). (Figure 2)

## Discussion

NE is a worldwide health problem frequently encountered in childhood. PMNE represents about 85% of these cases and causes substantial psychological distress for both children and their families [2]. The currently recommended treatment is not effective in all cases with a significant rate of relapse [5]. This prospective randomized study was done to assess the efficacy of PTNS versus desmopressin as two modalities in the treatment of PMNE, to find a more effective and durable treatment than the currently recommended therapy.

This study demonstrated that both PTNS and Desmopressin are effective in the treatment of patients with PMNE but, unfortunately, with a high rate of relapse, suggesting the need for maintenance therapy.

The results of this study were comparable to those of Capitanucci et al. [20], which revealed that PTNS could be applied quickly and safely in children as a single weekly session makes PTNS more compliant than a daily dose of medical treatment with its side effects [20].

To our knowledge, few prospective trials have studied the effectiveness of this treatment modality in such cases. Still, it has been successfully used for other cases like Refractory PMNE, which was described by Raheem et al. [14]. In this study, 78.6% of patients improved in response to PTNS, which was comparable to our results, in which 77.5% of children were responders [14].

Another use for PTNS was overactive bladder syndrome, as described by Peter et al. [21], in which there was a significant improvement in the PTNS group than the sham group (54.5% vs 20.9%, respectively) [21]. Also, PTNS is used with high success rates for the treatment of children with non-neurogenic lower urinary tract dysfunctions (60–80% of children with overactive bladder and 43–71% of children with urinary retention), as shown by Hoebeke et al. [22] (Table 3).

**Table 3.** Studies conducted on PTNS.

Author	Year	Total Number of patients	Number Of PTNS group	Complain	The intervention group	The Control group	Response in PTNS group	Methodology
Elshafey	2015	80	40	PMNE	PTNS + medical treatment	Alarm + medical treatment	87.5%	Prospective
Raheem	2013	28	14	Refractory PMNE	PTNS + behavioral therapy	Placebo + behavioral Therapy	87.6%	Pilot study
Peter	2010	220	110	Overactive bladder	PTNS	Sham therapy	54.5%	Prospective
Carlo	2017	105	35	Overactive bladder	PTNS	2 groups (Solifenacin and combination)	Significant improvement (percent not Mentioned)	Prospective
This Study	2020	80	40	PMNE	PTNS + behavioral therapy	Desmopressin + behavioral therapy	77.5%	Prospective

On the other hand, this study found that desmopressin was also effective in treating children with PMNE. This was comparable to results of previous studies like the Cochrane systematic review done by Glazener et al. [6] in which 47 RCTs involving 3448 children (of whom 2210 received Desmopressin) who found that Desmopressin was effective in reducing bedwetting and children have 1.34 fewer wet nights per week [6].

Unfortunately, in this study, we found a high relapse rate after 1 month of stopping PTNS, suggesting that PTNS may have temporary efficacy in treating PMNE and that its effect decreases gradually with time. This finding was also noted in cases with overactive bladder treated with PTNS, as shown by van der Pal [23]. Also, this was the same result found by Raheem et al. [14].

A high rate of relapse was also found in this study after 1 month of follow-up post-desmopressin treatment. This agreed with several studies that reported relapse of NE after discontinuation of Desmopressin, as Bayne et al. [24] and Alloussi et al. [25] that reported relapse rates between 80% and 100% after discontinuation of Desmopressin [24,25]. The lower relapse rate recorded in our study (48%) may be due to tapering the dose of desmopressin rather than abrupt discontinuation.

In this study, we found that there was no statistically significant difference between relapse rate between both groups (PTNS vs desmopressin groups)

We noticed that PTNS and Desmopressin are viable treatment options for patients with PMNE. However, using PTNS or desmopressin should be individualized. PTNS is more expensive and requires a weekly visit to the clinic which may be a burden on the parents; the patient must spend half an hour at the clinic to complete the session, which may be a burden on the doctor; some children refuse it from the start when they see the electrode’s needle. Desmopressin may be uncomfortable in some children who cannot swallow tablets, some children may be less compliant due to the daily regimen, or it may be contraindicated in some children. So, we should tailor the treatment according to the child and caregiver’s condition.

The main limitations of our study were the short one-month follow-up and the fact that we did not test the placebo effect. However, the aim of the study was to compare two established modes of treatment.

We recommend further studies to determine the long-term effects of posterior tibial nerve stimulation on mono-symptomatic nocturnal enuresis and specific protocols for maintenance therapy.

## Conclusion

Both PTNS and desmopressin are viable treatment options in patients with PMNE. PTNS is a good option if desmopressin is contraindicated, fearful of its side effects, or ineffective. We should tailor the treatment according to the child and caregiver’s condition. Relapse in some responders in both groups with time suggests the need for maintenance therapy.

## List of abbreviations

PTNS: Posterior Tibial Nerve Stimulation
PMNE: Primary Mono-symptomatic Nocturnal Enuresis
NE: Nocturnal Enuresis
REC: Research Ethics Committee
SPSS: Statistical Package for the Social Sciences
RCTs: Randomized Controlled Trials
OAB: Over Active Bladder
